# Coronary Thrombosis without Dissection following Blunt Trauma

**DOI:** 10.1155/2016/8671015

**Published:** 2016-02-23

**Authors:** Archana Sinha, Michael Sibel, Peter Thomas, Francis Burt, James Cipolla, Peter Puleo, Keith Baker

**Affiliations:** ^1^Division of Cardiovascular Disease, Saint Luke's University Health Network, Bethlehem, PA 18015, USA; ^2^Department of Emergency Medicine, Saint Luke's University Health Network, Bethlehem, PA 18015, USA; ^3^Department of Trauma Surgery, Saint Luke's University Health Network, Bethlehem, PA 18015, USA

## Abstract

Blunt trauma to the chest resulting in coronary thrombosis and ST elevation myocardial infarction (STEMI) is a rare but well-described occurrence in adults. Angiography in such cases has generally disclosed complete epicardial coronary occlusion with thrombus, indistinguishable from the findings commonly found in spontaneous plaque rupture due to atherosclerotic disease. In all previously reported cases in which coronary interrogation with intravascular ultrasound (IVUS) was performed in association with acute revascularization, coronary artery dissection was implicated as the etiology of coronary thrombosis. We present the first case report of blunt trauma-associated coronary thrombosis without underlying atherosclerosis or coronary dissection, as documented by IVUS imaging.

## 1. Introduction

Cardiac injury is an uncommon complication of blunt trauma; in most cases of cardiac dysfunction following blunt trauma injury, cardiac contusion is implicated. Arrhythmic death has been reported in children following blunt force injuries to the chest. In rare cases, coronary thrombosis and STEMI have been associated with blunt trauma to the chest in adults [1–3]. Angiography in such cases has generally disclosed complete epicardial coronary occlusion with thrombus, indistinguishable from the findings commonly found in spontaneous plaque rupture due to atherosclerotic disease [1–3]. In all previously reported cases in which coronary interrogation with IVUS was performed in association with acute revascularization, coronary artery dissection was implicated as the etiology of coronary thrombosis [4–8]. We present the first case report of blunt trauma-associated coronary thrombosis without underlying atherosclerosis or coronary dissection, as documented by IVUS imaging performed at the time of primary percutaneous revascularization.

## 2. Case Report

**Figure 1:**
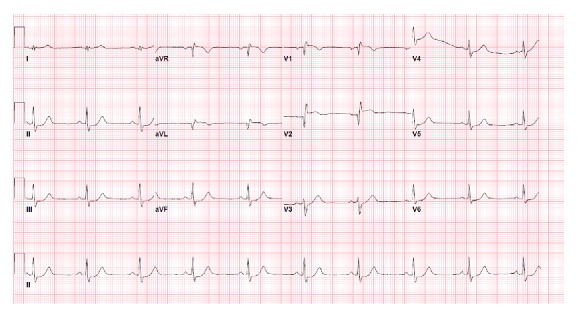
EKG showing ST segment elevation in leads V1, V2, and aVL and ST segment depressions in leads II, III, aVF, V5, and V6.

**Figure 2:**
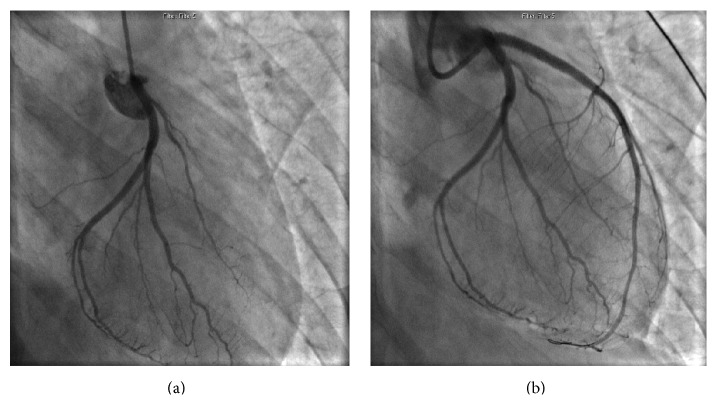
(a) Coronary angiogram showing total occlusion of LAD. (b) Restoration of normal flow following balloon dilation and stent deployment.

**Figure 3:**
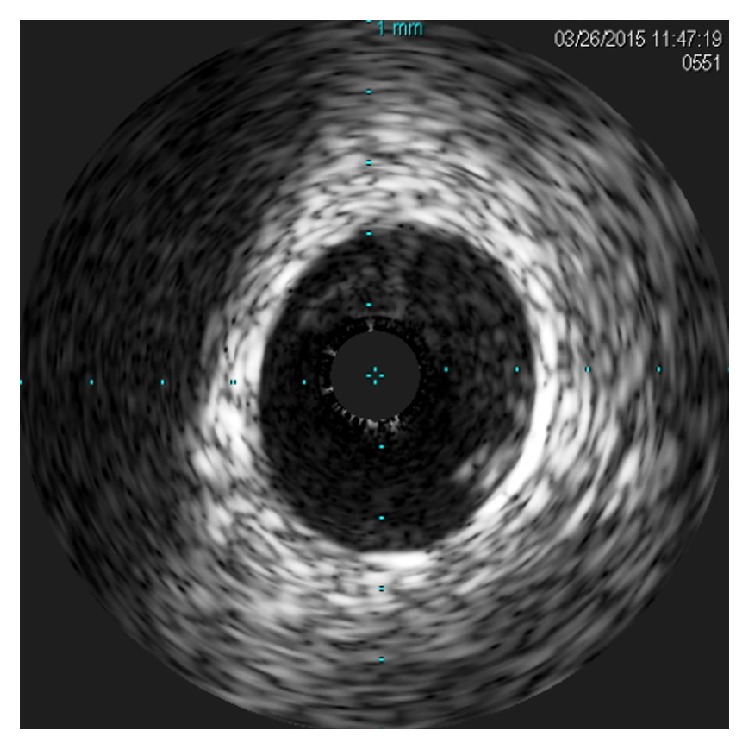
Intravascular ultrasound (IVUS) showing thrombus in proximal LAD, no evidence of dissection.

A 25-year-old previously healthy woman presented to the Emergency Department after sustaining a kick to her lower chest by a horse. She initially complained of epigastric pain and nausea; she also reported substernal chest discomfort that became progressively more prominent. She had no coronary atherosclerotic risk factors and did not use oral contraceptives. She had stable vital signs; physical examination was notable only for abrasions of the chest wall. An ECG revealed sinus bradycardia at 56 beats/min with ST segment elevation in leads V1, V2, and aVL and ST segment depressions in leads II, III, aVF, V5, and V6 (Figure 1). CT imaging of the chest, abdomen, and pelvis disclosed a small left pneumothorax, focal myocardial hypoattenuation suggesting the possibility of cardiac contusion, and soft tissue attenuation of the subhepatic porta hepatis indicating hemorrhage. An echocardiogram demonstrated severe hypokinesis of the mid and distal anteroseptal segments and the apex, with an estimated left ventricular ejection fraction of 45%. The aortic root was without evidence of dissection; there was no pericardial fluid or aortic valvular insufficiency. Because of the ECG and echo findings, emergent cardiac catheterization was performed. Coronary angiography via the right radial artery disclosed a total occlusion of the left anterior descending artery 2 mm from its origin (Figure 2(a)). All other epicardial vessels appeared to be normal. Antiplatelet and antithrombotic therapies with aspirin, clopidogrel, and bivalirudin were administered. A coronary wire was passed to the distal LAD, and balloon dilation with an undersized 2.5 mm balloon was performed, restoring normal antegrade flow. IVUS was then performed. Thrombus was evident adherent to the vessel wall in the proximal LAD. No dissection or atherosclerotic plaque was present (Figure 3/Video in Supplementary Material, available online at http://dx.doi.org/10.1155/2016/8671015). A 4.0 × 23 mm everolimus-eluting stent was deployed across the site of total occlusion (Figure 2(b)). Repeat IVUS imaging disclosed a lack of full stent apposition, and postdilation was performed with a 4.5 mm noncompliant balloon. Following PCI, antegrade flow was normal. CT angiography disclosed no evidence of aortic dissection. The patient exhibited mild pulmonary congestion on the following day that responded to diuretic therapy. Maximum serum troponin I level was 69.1 ng/mL. Hypercoagulability evaluation, including homocysteine, anti-nuclear antibody screen, antithrombin III, protein C, free and total protein S, protein S activity, Factor V Leiden mutation analysis, prothrombin 20210GA mutation, lupus anticoagulant profile, and anticardiolipin antibody, was negative. She was discharged on the 5th hospital day. Repeat echocardiography 2 weeks after her initial injury showed improvement in LV function, with residual mild to moderate hypokinesis of the mid to apical anterior wall and an ejection fraction of 50%.

## 3. Discussion

A wide variety of cardiovascular pathologies have been associated with blunt trauma injury, including myocardial contusion, aortic transection or less often aortic dissection, trauma-induced ventricular arrhythmia or commotio cordis in young children [9], hemopericardium with tamponade, and aortic valve leaflet avulsion [10–12]. Acute myocardial infarction (AMI) is a rare but well-described complication of blunt trauma to the chest. Since the advent of primary percutaneous intervention, there have been several reports of AMI due to total epicardial thrombotic occlusion following blunt trauma and its successful treatment by percutaneous intervention [13–17]. Proposed mechanisms for AMI in this setting have included intimal injury due to shear forces imparted by the blunt trauma [18, 19], plaque rupture, coronary artery dissection, and coronary vasospasm [20]. In several reports, the presence of coronary artery dissection has been documented as the underlying pathophysiologic trigger for thrombosis [4–8]. In recent years, IVUS has become an increasingly routine adjunctive imaging modality in coronary revascularization. For a 40 MHz IVUS transducer the typical resolution is 80–100 microns axially and 200–250 microns laterally [21]. IVUS is extremely sensitive for the detection of coronary disease involving the vessel wall, including the presence of underlying atherosclerosis or dissection. All previous reports of blunt trauma-associated acute STEMI that included IVUS interrogation of the occluded epicardial vessel have identified the presence of a coronary dissection as the underlying pathophysiologic substrate triggering thrombosis. Coronary dissection presumably occurs in association with deceleration trauma, in some cases involving the aorta. It is not known whether affected individuals carry a genetic predisposition to vascular dissection; however, spontaneous coronary dissection has been associated with fibromuscular dysplasia [22]. The mechanism of thrombosis in the case presented here remains obscure. Acute psychological stress has been implicated in AMI, following diverse stressors, including earthquakes [23], missile attacks [24], and international soccer matches [25], but these primarily involved patients having underlying atherosclerotic disease, whereas the patient in the current case was a 25-year-old female with no coronary risk factors and no atherosclerosis by angiography or by IVUS interrogation of the vessel wall. It appears most likely that thrombosis in this case was the result of endothelial injury, possibly caused by direct compression of the proximal left anterior descending artery by the force of the blow or via shock waves [26]. A mathematical model for blunt injury leading to hemodynamic shade zone formation with high and low shear stress and hyperviscosity has been developed by Ismailov [27]. In addition, the presence of an underlying subclinical thrombophilic state that might interact with such an injury to produce thrombosis cannot be excluded. Park et al. demonstrated that patients with blunt trauma have greater numbers of circulating procoagulant microparticle and increased in vitro thrombin generation that correlated with injury severity despite normal values for standard clotting assays. Response to injury though appears to be variable between individuals [28].

The treatment approach to myocardial infarction (STEMI) following blunt chest trauma is immediate coronary angiography and revascularization with percutaneous intervention and continuation of dual antiplatelet therapy. We felt that use of GpIIb/IIIa inhibitor was contraindicated in our patient because of liver laceration and increased risk of bleeding.

In conclusion, we present a case of proximal left anterior descending artery thrombosis precipitated by blunt force trauma to the chest in a 25-year-old woman with no history or risk factors for coronary disease and with no associated atherosclerosis or coronary artery dissection. The patient was successfully treated by primary PCI. Although significant chest pain would not be unexpected following severe blunt trauma injury to the chest, the possibility that ongoing pain represents myocardial ischemia should be considered, and a screening ECG should be considered to identify rare cases of STEMI.

## Supplementary Material

IVUS pullback from the proximal LAD into the left main artery. The arterial wall is normal, with no evidence of atherosclerotic plaque or dissection. A crescent of thrombus is seen at the top of the lumen of the proximal LAD in the first frames of the video image. With pullback proximally to the LAD ostium, the thrombus is seen to occupy most of the LAD lumen around the IVUS catheter. No thrombus is seen within the lumen of the left main artery.
